# Pesticide residue levels in vegetables and surface waters at the Central Rift Valley (CRV) of Ethiopia

**DOI:** 10.1007/s10661-020-08452-6

**Published:** 2020-07-27

**Authors:** Kumelachew Mulu Loha, Marja Lamoree, Jacob de Boer

**Affiliations:** Department of Environment & Health, Vrije Universiteit, De Boelelaan 1085, 1081 HV Amsterdam, The Netherlands

**Keywords:** Pesticide residue, Central Rift Valley, Maximum residue limits (MRLs), Vegetables, Surface waters

## Abstract

Seven pesticides, profenofos, metalaxyl, λ-cyhalothrin, 4,4′-DDT, 4,4′-DDE, and α- and β-endosulfan, were determined in vegetables (tomato, onion) from 20 locations and surface waters from 12 locations in the Central Rift Valley (CRV) of Ethiopia. Quick, Easy, Cheap, Effective, Rugged, and Safe (QuEChERS) and solid phase extraction (SPE) methods were used for the vegetables and water, respectively. In 2.5% of the tomato samples, profenofos was detected above European maximum residue limits (MRLs), in 12.5% of the samples metalaxyl, and in 2.5% α- and β-endosulfan. In 5% of the onion samples, profenofos was detected above European MRLs, in 7.5% of the onion samples metalaxyl, and in 5% λ-cyhalothrin. In surface water, profenofos was detected at the highest concentration of 2300 μg/L in the Bulbula River, 890 μg/L near the agricultural land north of Lake Ziway (ANLZ-1), 1700 μg/L in the floriculture effluent (FE-1), and 900 μg/L in tap water at the Batu Drinking Water (BDW) supply. These results show that the levels of pesticides are in several cases substantially elevated, and emphasize the need of regular pesticide monitoring programs for surface waters and vegetables in the Ethiopian CRV.

## Introduction

Worldwide, population increase results in a need for the production of more food (Akoto et al. 2015). Agriculture is the main source of income for more than 80% of the population of Ethiopia. During the last couple of years, large-scale floriculture farms have been expanding in many parts of the country (Ethiopian Investment Agency 2012), and floriculture industry became the major source of economic development with a substantial export of 714.5 million cut flowers and 49,000 tons of roses in 2016 which is a 10.7% increase compared with 2015 (Agarwa et al. 2010; Ethiopian Press Agency and Floral Daily 2016; Srivastava et al. 2011). Excessive application and improper handling of pesticides affect the environment and cause serious health problems to human beings and animals. Organochlorine pesticides are persistent and bio-accumulate in the environment (Akca et al. 2016; Fiedler et al. 2013). They cause ecological problems and have a great impact on human health (Agarwal et al. 2015; Fiedler et al. 2013). Although most organochlorine pesticides were banned in 2004 (Stockholm Convention 2001), DDT is still used indoors in Ethiopia to control the spread of malaria (Jansen and Harmsen 2011; Mengistie et al. 2017). Local farmers sometimes use DDT illegally for agricultural purposes, and Ethiopia registered endosulfan to control the damage caused by cotton pests; however, its use is also reported for vegetable and fruit pests (Mengistie et al. 2017). Due to improper application of pesticides, many vegetables contain pesticide residues above their maximum residue limits (MRLs) (Bhanti and Taneja 2005). Too high levels of pesticides also reduce the expected yield. Hence, it is essential to monitor and control pesticide residue level in crops and vegetables (Handford and Campbell 2015). Different African countries established MRLs based on toxicological and agronomic studies (D’Mello 2003). Ethiopia is the so-called water tower of the East-African counties because the country has quite a number of natural and artificial lakes. Most of these lakes are located in the Rift valley, and they are often used for fish production (Teklu et al. 2015). As a result of exhaustive agricultural activities, nutrients and agrochemicals accumulate in soil, lakes, rivers, ponds, and water drainage channels and they become a major ecological problem (Agarwal et al. 2010; Sneha and Bhimte 2012).

The Quick, Easy, Cheap, Effective, Rugged, and Safe (QuEChERS) method was developed in 2003 (Anastassiades et al. 2003) and is an efficient and convenient extraction technique for the analysis of pesticides and other contaminants in agricultural products. This method has been used widely to analyze pesticide residues in tomatoes (Angioni et al. 2011; Lehotay et al. 2010).

Pesticide residues have been reported frequently in fruits, vegetables, and water in many countries including Ethiopia (Diop et al. 2016; Fernandes et al. 2011; Lu et al. 2012; Mutengwe et al. 2016; Ozcan 2016; Tadeo and Sanchez-Brunete 2003). However, there is very little information about pesticide residues in samples from the Central Rift Valley (CRV) region of Ethiopia. Therefore, the present study was undertaken to determine pesticide residues in tomato, onion, and water samples from this region.

## Materials and methods

### Description of study areas

**Fig. 1:**
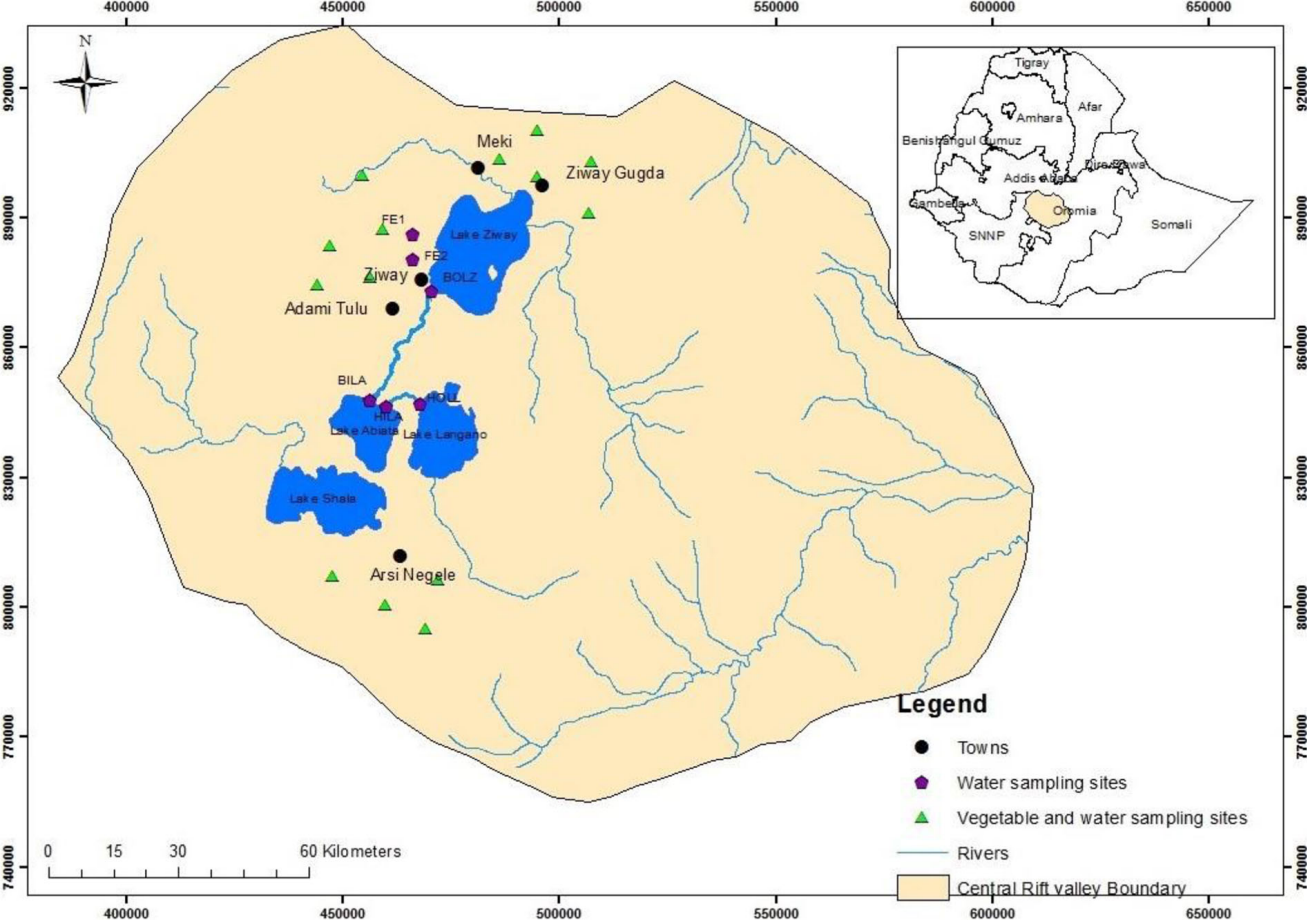
Sampling locations in the CRV for vegetable and water samples (Bulbula and Horankelo rivers)

This study was conducted in the CRV of Ethiopia, which is located approximately between 38° 05′ E and 39° 25′ E, and between 7° 06′ N and 8° 27′ N. The Oromiya regional state government administration of Ethiopia divided this valley into two main administrative districts (*Woredas*). The low land areas, which are located in the Rift floor, have four administrative districts, namely Adamitulu Judo Kombolcha (AJK), Dugda Bora (DB)/(Meki), Arsi Negele (AN), and Ziway Gugda (ZG). The high land areas have six administrative districts, namely Sodo, Mekana, Mareko, Tiyo, Degeluna Tiyo, and Munessa. Tomatoes, onions, green peppers, and cabbage are largely grown at the Rift floor, whereas potatoes, carrots, beet root, garlic, and sugar cane are grown at the high land areas (Scholten 2007). At the Rift floor, there are four lakes (Ziway, Langano, Abiata, and Shala) and four rivers (Meki, Ketar, Bulbula, and Horankelo) (Fig. 1). The Ketar and Meki rivers are the main rivers entering Lake Ziway, i.e., the Meki river from the plateau west of Lake Ziway and the Ketar river from the eastern and south-eastern plateaus. The Bulbula River connects Lake Ziway (upstream) and Abiata (downstream), and a major part of the water for Abiata comes from Lake Ziway. Therefore, these two lakes are hydrologically connected. The Horankelo River connects this lake and Lake Langano. Both Lake Abiata and Lake Shala are lakes without surface water outflow (Alemayehu et al. 2006; Ayenew 2004; Jansen et al. 2007). The CRV of Ethiopia is situated in the tropical zone, and its maximum temperature varies from 25 to 29 °C during the short rainy season (locally known as *Belg*). During the main rainy season (locally known as *Kiremt*), the maximum temperature varies from 22 to 26 °C (Kassie et al. 2012). The climate of the lowlands surrounding the lakes is arid and semi-arid, and the highlands are humid to dry sub-humid (Scholten 2007).

### Chemicals and apparatus

For the extraction and clean-up of the samples, isooctane, n-hexane, dichloromethane, ethyl acetate, methanol, acetonitrile, sodium citrate dihydrate, sodium hydrogen citrate sesquihydrate, sodium chloride, magnesium sulfate, and formic acid and QuEChERS dispersive tubes containing 0.15 g primary secondary amine (PSA) and 0.9 g magnesium sulfate were used. Profenofos, metalaxyl, λ-cyhalothrin, 4,4′-DDT, 4,4′-DDE, and α- and β-endosulfan were used as native pesticide standards procured from Chiron (Trondheim, Norway). The internal standards (ISTD) used were the following: metalaxyl-D6 was purchased from Toronto Research Chemicals (TRC, Toronto, Canada); 4,4′-DDE (ring-^13^C_12_), α-endosulfan D_4_, and β-endosulfan D_4_ were purchased from Cambridge Isotope Laboratories, (Andover, USA); TPP (triphenyl phosphate) and permethrin were purchased from Sigma-Aldrich Chemie N.V (Schnelldorf, Germany); and OASIS HLB cartridges were procured through Waters Chromatography B.V. (Etten-Leur, The Netherlands).

### Chromatographic analysis

Analysis was performed using an Agilent 6890 GC coupled to a 5975 mass spectrometer (MS) under the following conditions: profenofos, metalaxyl, λ-cyhalothrin, 4,4′-DDT, and 4,4′-DDE were analyzed using electron impact (EI) MS and a DB-5MS gas chromatographic (GC) column (Agilent no. 122–5532), 30 m length, 250 μm internal diameter (ID), 0.25 μm film thickness. The carrier gas used was helium at a flow rate of 1.0 mL/min. The injection volume was 1 μL in the splitless mode at 275 °C. The GC oven was programmed as follows: initial temperature 90 °C, hold for 2 min then ramped at 20 °C/min to 170 °C followed by a ramp of 5 °C/min to 310 °C and hold for 14.33 min. α- and β-Endosulfan were analyzed using electron-capture negative chemical ionization (ECNI) MS and a RTX-1614 GC column (Restek 10296), 15 m length, 250 μm I.D., and 0.1 μm film thickness. Helium was used as a carrier gas at a flow rate of 1.0 mL/min. The injection volume was 1 μL in the splitless mode at 275 °C. The GC oven was programmed as follows: initial temperature 90 °C, hold for 3 min, then ramped at 5 °C/min to 310 °C, and hold for 8 min.

### Sample collection and preparation

#### Vegetable samples

For vegetable (tomato and onion) sampling, four areas at the Rift floor were selected based on the potential for tomato and onion production. To collect these samples, one farm land was randomly selected from each of the sub-administrative districts (*Kebeles*), and they were sampled during two sampling campaigns in 2015. The first sampling campaign was in winter, in the beginning of March, and the second one was in spring at the end of May. The first campaign was during the beginning of short rainy season, whereas the second one was during the main rainy season at CRV (Kassie et al. 2012). From each farm land and sampling campaign, composite samples of 2 kg tomatoes and 2 kg onions were collected separately.

The samples were homogenized by a heavy-duty blender (Waring Commercial, 1500 W, Homogenizer, USA), and 200 g of each of the composite samples was transferred into a sampling bottle and was kept for 3 weeks in the refrigerator at 4 °C. Then, they were packed in an ice box and were transported in frozen condition to The Netherlands for analysis.

#### Water samples

Surface water was collected in the CRV region from four categorized areas, namely water bodies (at the inlets and outlets of the lakes), agricultural lands, floriculture effluents, and drinking tap water (Fig. 1). The samples were collected from the same 12 locations during four sampling campaigns in March, May, and July 2015 and in July 2016. The water sampling locations were Horankelo River outlet from Lake Langano (HOLL), Horankelo River inlet to Lake Abiata (HILA), Bulbula River Outlet from Lake Ziway (BOLZ), and Bulbula River inlet to Lake Abiata (BILA). For agricultural lands, the sampling locations were agricultural land north of Lake Ziway (ANLZ-1, ANLZ-2, and ANLZ-3) and agricultural land along Lake Ziway (AALZ-1 and AALZ-2). The effluent waters from the floriculture enterprises at Ziway area, floriculture effluents (FE-1 and FE-2), and drinking water samples were collected from tap at Batu (Ziway) Drinking Water (BDW). In each of the sampling campaigns, in total 3 L (12 × 0.25 L) of surface waters was collected using bottles which were previously cleaned and rinsed by acetone and completely dried. To remove turbidity and debris, samples were filtered through glass fiber filters and stored at 4 °C prior to extraction and were transported to The Netherlands for laboratory analysis.

### Extraction method and clean-up of the extracts

#### QuEChERS extraction

A QuEChERS method with slight modification was used for the extraction of tomato and onion samples (Anastassiades et al. 2003; EN 15662 2008). The method is summarized as follows: 10 g of each tomato and onion sample was weighed in a 50-mL centrifuge tube and was spiked with 100 μL of the ISTD, mixed, and left to stand for 10 min at room temperature before extraction. Then, 10 mL of acetonitrile was added to each of the two mixtures and was vortexed for 1 min. Then, citrate buffer containing 1 g sodium citrate dihydrate, 0.5 g sodium hydrogen citrate sesquihydrate, 1 g sodium chloride, and 4 g magnesium sulfate was added, and immediately shaken for 1 min, followed by centrifugation for 5 min at 2000 rpm. The clean-up process for both samples was performed using QuEChERS dispersive tubes. Then, 6 mL of the upper extract was transferred to this dispersive tube and was shaken for 30 s followed by 5-min centrifugation at 2000 rpm. Then, 3 mL of the supernatant was transferred into an evaporating tube and immediately acidified with 40 μL of 5% formic acid in acetonitrile to avoid the degradation of pesticides sensitive for high pH. Both extracts were evaporated under a stream of N_2_ at a temperature between 30 and 40 °C and were reconstituted in isooctane until the final volume became 100 μL.

#### Solid phase extraction

A solid phase extraction (SPE) method was used to extract the target pesticides from water samples with a slight modification (Kouzayha et al. 2012). The method is described as follows: (1) An OASIS HLB cartridge was conditioned with 3 mL n-hexane, 3 mL dichloromethane, 3 mL ethyl acetate, and 3 mL methanol, respectively, and was equilibrated with 3 mL of MilliQ water without allowing the cartridge to dry out. (2) The collected water sample was passed through the conditioned cartridge at a flow rate of 4 mL/min. (3) The cartridge was dried for 30 min under vacuum. (4) The sample was washed with 3 mL of 5% methanol in water. (5) The analyte was eluted from the solid phase with 3 mL of n-hexane, 3 mL dichloromethane, and 3 mL ethyl acetate, respectively. (6) The extract was evaporated to dryness under a stream of N_2_ and the residue was reconstituted with 100 μL of isooctane and was transferred to an auto-sampler vial for GC analysis.

### Method validations

#### Recovery studies

For the recovery studies of the QuEChERS extraction, 2 kg pesticide-free tomatoes was procured and homogenized with a blender. Then, 200 g was taken for each of the seven replicates, and one blank solution as a control. A total of 100 μL of ISTD mixture was added to each of the replicates and blank. The spiking mixture containing all target pesticides had a concentration of 7.5 μg/L in isooctane per pesticide. The extraction and clean-up process were performed according to the method described earlier. Finally, the recovery was calculated, and the relative standard deviation (RSD) was obtained. For the recovery study of the SPE method, 2.5 L surface water was collected and spiked with 100 μL of a spiking solution containing all target pesticides in a concentration of 350 μg/L in isooctane. The spiked water solution was stirred for 18 h and approximately 250 mL of this water was transferred into ten screw cup reagent flasks. This study was conducted in eight spiked replicates, one blank, and one non-spike water sample, and 100 μL ISTD mixture was added. The SPE method was also described earlier. The theoretical concentration was calculated, and the real concentration on a wet weight basis was obtained from the experimental result. All data were analyzed and calculated using MSD Chemstation G170DA D.02.00.275 and finally the recovery of each pesticides was calculated.

#### Calibration studies

Solvent calibration solutions (SC) containing all target pesticides were prepared in isooctane at six concentration levels. A total of 1 μL of this level solution was injected into the GC/ECNI-MS for α- and β-endosulfan, and the rest of the pesticides were injected into GC/EI-MS, each time starting with the lowest calibration concentration.

## Results and discussion

### Analytical method validations

#### Vegetable samples

The mean recoveries of all pesticides in tomato were between 73.2 and 95.7%. The results were in the acceptable analytical range from 70 to 120% (Berrada et al. 2010; Osman et al. 2010). The RSD was also below the commonly accepted level (< 20%) (Table 1) except for α-endosulfan (24.1%). These results meet the requirements of European Commission document no. SANCO/12495/2011 (SANCO 2011). Therefore, the QuEChERS method is considered appropriate to achieve results for the pesticides analyzed in this study.

**Table 1:** Mean recovery (mean %*R*) and RSD of the testing methods in tomato and water at a spiking level of 7.5 μg/L and 350 μg/L, respectively

Target pesticides	Tomato		Water	
	Mean %*R*	RSD	Mean %*R*	RSD
Profenofos	80.4	15.4	52.3	5.7
Metalaxyl	82.6	10.1	87.4	13.1
λ-cyhalothrin	73.2	6.0	83.9	11.0
4,4′-DDT	95.7	6.1	126.1	22.5
4,4′-DDE	87.3	10.3	106.9	15.7
α-Endosulfan	91.1	24.1	110.9	5.7
β-Endosulfan	93.2	18.0	97.0	3.1

The linearity of the analytical method for tomato and onion is shown in Table 2. Correlation coefficients (*r*^2^) were good with 0.981 and 0.979, respectively. The LOD was calculated as three times the noise height and the LOQ is 3.3 times the LOD. For tomato, the LOD and LOQ varied from 0.004 to 0.27 μg/kg and 0.012 to 0.89 μg/kg, respectively, and for onion, these varied from 0.001 to 0.094 μg/kg and 0.004 to 0.31 μg/kg, respectively (Table 2).

**Table 2:** Limits of detection (LOD), limits of quantification (LOQ), and linearity of the testing methods in tomato, onion, and water

Target pesticides	Tomato			Onion			Water		
	Linearity	LOD	LOQ	Linearity	LOD	LOQ	Linearity	LOD	LOQ
	(*r*^2^)	μg/kg		(*r*^2^)	μg/kg		(*r*^2^)	μg/li	
Profenofos	0.998	0.270	0.891	0.990	0.060	0.196	0.998	0.029	0.095
Metalaxyl	0.996	0.004	0.012	0.979	0.033	0.109	0.986	0.070	0.229
λ-Cyhalothrin	0.981	0.032	0.106	0.996	0.094	0.312	0.996	0.025	0.083
4,4′-DDT	0.998	0.029	0.097	0.981	0.057	0.187	0.984	0.002	0.007
4,4′-DDE	1.000	0.074	0.245	0.988	0.017	0.057	0.989	0.050	0.165
α-Endosulfan	0.999	0.006	0.019	0.994	0.001	0.004	0.988	0.001	0.004
β-Endosulfan	0.998	0.012	0.040	0.993	0.005	0.016	0.986	0.016	0.052

#### Water samples

The mean recoveries of the pesticides in water ranged between 83.9 and 110.9%, which is within the acceptable analytical range (70–120%) except for profenofos (52.3%) and 4,4′-DDT (126.1%) (Table 1). Their RSD values were below 20%, except for 4,4′-DDT (22.5%). This shows the performance in this validation is in line with the commonly accepted level (RSD < 20%), while the determination of DDT was more difficult, probably due to occasional decomposition during GC analysis.

The linearity of the analytical method for water is shown in Table 2. The calibration curve results were shown as a correlation coefficient (*r*^2^). The lowest value (*r*^2^ = 0.984) was recorded for 4,4′-DDT and the highest (*r*^2^ = 0.998) for profenofos. This indicates a very good linearity of the calibration curve. LOD and LOQ results are shown in Table 2. The LOD ranged from 0.001 to 0.070 μg/L and the LOQ from 0.004 to 0.229 μg/L.

### Pesticide residues in tomato samples

Table 3 summarizes the pesticide concentration in tomato samples during March and May of the 2015 sampling campaigns.

**Table 3:** Pesticide concentrations (μg/kg) in tomato samples (on a wet weight basis) collected from CRV of Ethiopia

Sampling area	Location name	Target Pesticide List													
		Profenofos		Metalaxyl		λ-Cyhalothrin		4,4′-DDT		4,4′-DDE		α-Endosulfan		β-Endosulfan	
		MRL in μg/kg													
		50		200				3500				50			
		The first (March) and second (May) sampling campaign of 2015													
		March	May	March	May	March	May	March	May	March	May	March	May	March	May
Adamitulu Judo Kombolcha (AJK)	Edo Gojola	29.0	14.0	19.0	31.0	8.1	ND	7.60	< 0.1	1.9	0.4	5.4	4.2	24.1	13.3
	Abune Germama	28.0	13.0	17.0	62.0	12.0	23.0	1.3	0.5*	3.20	1.3	6.1	5.8	24.2	10.6
	Bocessa	15.0	12.0	14.0	67.0	9.3	1.6	1.0	0.6*	1.5	< 0.6	2.4	1.6	9.7	3.0
	Doodicha	25.0	9.4	14.0	110	1.30	77.0	1.1	< 0.2	1.4	0.7	7.6	3.2	20.7	8.0
	Ilika Chelema	14.0	13.0	13.0	89.0	4.5	3.3	12.0	0.2	2.5	0.4*	3.7	0.9	11.4	1.3
Dugda Bora (DB	Welda Mekedela	21.0	7.5	250	16.0	4.0	71.0	0.5	< 0.1	0.9	0.7	6.2	2.9	18.3	6.8
	Welda Kelina	26.0	9.2	**560**	15.0	12.0	12.0	0.8	0.4*	2.1	0.4*	8.1	3.4	25.3	8.6
	Shubi Gamo	11.0	0.8	14.0	1.1	11.0	16.0	0.9	0.5*	1.3	< 0.5	2.3	2.3	9.9	5.9
	Bekeli Girisa	42.0	26.0	**240**	110	40.0	96.0	0.7	< 0.3	1.0	< 0.4	7.5	7.5	38.1	8.9
	Garba Korke Adi	11.0	0.7	13.0	19.0	21.0	8.6	0.8	< 0.7	1.2	< 0.9	2.8	3.9	12.4	12.7
Arsi Negele (AN)	Shorba	15.0	21.0	14.0	**380**	110	19.0	< 0.2	< 0.1	0.6*	1.1	4.3	5.7	12.1	17.7
	Hargeda	8.1	24.0	13.0	13.0	0.3	14.0	0.6*	0.4*	0.7*	0.4*	3.1	6.7	5.3	19.5
	Kerara	8.2	11.0	74.0	110	1.7	3.0	0.6	2.1	0.5	0.4	29.0	0.6	34.0	0.9
	Gedemso	**64**	20.0	16.0	**510**	0.1	25.0	7.6	1.6	0.5*	0.4	19.7	5.4	57.0	15.7
	Turge	ND	18.0	ND	180	ND	2.0	ND	0.4	ND	0.5	**1600**	6.7	**690**	18.5
Ziway Gugda (ZG)	Burka Lemefa	0.8	14.0	2.3	90.0	5.5	6.9	*0.2	< 0.3	0.5*	< 0.4	2.1	5.2	5.4	19.0
	Sengo	14.0	28.0	2.0	150	8.1	1.8	1.9	1.2*	< 0.3	< 0.6	2.9	5.8	11.2	18.4
	Galbe	1.0	31.0	1.3	21.0	7.1	65.0	< 0.3	1.9	< 0.4	< 0.5	6.5	4.1	9.2	13.5
	Shenen Meja	1.4	40.0	2.6	160	0.7	< 0.1	2.2	0.4*	1.2*	< 0.2	0.5	5.8	0.5	19.6
	Burka Dalecha	0.9	0.4	13.0	10.0	0.1*	74.0	< 0.3	< 1.3	0.6*	< 1.6	1.3	2.6	3.3	8.1

*Value between LOD and LOQ, *< X* value less than LOD (*X*), *ND* not detected

Values were bolded just to show that they are significantly high results recorded among all the values obtained for the studied matrix

#### Profenofos

In the first sampling campaign, pronounced levels of profenofos were detected for most of the locations in the AJK and DB sampling areas due to the non-judicious application of this pesticide (Table 3). These two areas are the major tomato-producing regions within the country. In the first sampling campaign, 2.5% of all samples analyzed was found to be above the MRL (50 μg/kg) recommended by the European Union (European Commission Regulation No, 2005). In Senegal, profenofos was detected in tomato samples at concentrations of 80 μg/kg (Diop et al. 2016), which is higher than the maximum result obtained in this study.

#### Metalaxyl

Detectable levels of metalaxyl were found in tomato samples from all locations except at Turge during the first sampling campaign (Table 3). Among all tomato samples analyzed, 15% and 10% of them had metalaxyl residues exceeding MRL (200 μg/kg) (European Commission Regulation No, 2005) in the first sampling and second campaign, respectively. These residues of metalaxyl exceeding the MRL might be the consequence of misuse of a mixture of formulations containing metalaxyl. The metalaxyl concentrations in this study were much higher than those found in Colombia and Spain with concentrations ranging between 10 and 30 μg/kg for Colombia (Arias et al. 2014), and between 10 and 40 μg/kg in Spain (Camino-Sanchez et al. 2011).

#### λ-Cyhalothrin

λ-Cyhalothrin was detected in all tomato samples tested except at Turge during the first sampling campaign, and at Edo Gojola during the second one (Table 3). None of the samples had residues above the European MRL (200 μg/kg) (European Commission Regulation No, 2005). However, a concentration close to the MRL was obtained at Shorba in the first sampling campaign, probably due to the low vapor pressure (0.0002 mPa) of λ-cyhalothrin, and its low volatilization into the atmosphere (Li-Ming et al. 2008). In all other sampling locations and campaigns, residues remained well below the prescribed safe limits. Probably, the farmers in these locations might get advice from the nearby agricultural professionals about pesticide application rates and spray based on their recommendations.

#### DDT and DDE

Except at Turge, 4,4′-DDT and 4,4′-DDE were detected in all tomato samples in the first sampling campaign (Table 3). Because, the first sampling campaign was at the start of the short rainy season in the CRV region (Kassie et al. 2012). Since the country allows the use of DDT for malaria control (Jansen and Harmsen 2011), farmers prefer to spray DDT indoors at this time (Mengistie et al. 2017). However, none of the residues was above the MRL (3500 μg/kg) (Codex Alimentarius Commission 2009). This may be attributed to the restrictions imposed on the use of DDTs for agricultural purpose (Jansen and Harmsen 2011). The ratio of 4,4′-DDT to 4,4′-DDE varied in different locations for both campaigns. The one with the higher ratio confirms a recent application of DDT (Kumar and Mukherjee 2012; Tavares et al. 1999). In India, 4,4′-DDT was found at a concentration of 0.006 ng/kg in tomatoes collected from the market, which is much lower than in this study, and 4,4′-DDE was not detected at all (Kumari et al. 2002).

#### Endosulfan

Endosulfan residues were present in all tomato samples (Table 3), and 2.5% of them were found above the MRL value (500 μg/kg) (European Commission Regulation No, 2005). A pronounced level of α- and β-endosulfan were obtained in the first sampling campaign at Turge. It could be that farmers at this location sprayed endosulfans indiscriminately far beyond the recommended rate and dose. Although the level of both endosulfans in tomato samples was not as high as at Turge, 300 μg/kg of α-endosulfan and 430 μg/kg of β-endosulfan concentrations were reported in Ghana (Essumang et al. 2008). However, 13,880 μg/kg of β-endosulfan residue was found in tomato samples collected from the Omdurman central market in Khartoum State, Sudan (Ahmed et al. 2017) which is 20-fold higher than in the present study.

The technical mixture ratio of α-endosulfan to β-endosulfan is 3:1 (Sutherland et al. 2002; Walse et al. 2003). However, this ratio is different in tomatoes from location to location in the CRV. For 70% of the tomato samples, this ratio was 1:3; for 25% of the samples, the ratio was 1:1; and for 5% of the samples, a 3:1 ratio was found. These ratio differences could be related to the hot weather conditions in the CRV. Since the vapor pressure of α-endosulfan (0.4 mPa) is higher than that of β-endosulfan (0.08 mPa) (Freixo et al. 2015), lower concentrations of α-endosulfan can be expected in most of the samples. Various studies reported the degradation of endosulfan isomers. Parm et al. (1991) reported the fate and interconversion of α-endosulfan, β-endosulfan, and endosulfan sulfate (ESS) on chickpea (*Cicer arietinum Linn*) in a subtropical environment. The result showed that α-endosulfan was less persistent than β-endosulfan and ESS. The percentage loss of each isomer was much higher than in the temperate region (Chopra and Mahfouz 1977). Interconversion of stereoisomers of endosulfan on chickpea crop under field conditions was also studied (Mukherjee and Gopal 1994). The results indicated that the α isomer was converted to ESS on chickpea leaves in large quantities, whereas that happened only to a minor extent for the β isomer. This indicates that the β isomer is more persistent than the α isomer. Also, on various plant surfaces, α-endosulfan degraded (or evaporated) more rapidly than β-endosulfan (Goebel et al. 1982). In the atmosphere, Shunthirasingham et al. (2010) suggested that the ratio of endosulfan α/β is higher due to the loss of β-endosulfan, while ESS was found the most persistent and stable product of all endosulfan isomers (Ghadiri 2001; Kathpal et al. 1997; Walse et al. 2003).

Ghadiri (2001) reported that the soil-water ratio and temperature affect the degradation rate of both isomers of endosulfan. The results showed that, under humid conditions and high temperatures, α-endosulfan concentrations in soil may decline rapidly, and β-endosulfan degradation would be slower. The change of the α-endosulfan/β-endosulfan ratio was also reported on soil and plants due to the higher conversion of α-endosulfan to ESS than β-endosulfan to ESS (Antonious et al. 1998).

### Pesticide residues in onion samples

Table 4 summarizes the pesticide concentration in onion samples during March and May of the 2015 sampling campaigns.

**Table 4:** Pesticide concentrations (μg/kg) in onion samples (on a wet weight basis) collected from CRV of Ethiopia

Sampling area	Location name	Target Pesticide List													
		Profenofos		Metalaxyl		λ-Cyhalothrin		4,4′-DDT		4,4′-DDE		α-Endosulfan		α-Endosulfan β-endosulfan	
		MRL in μg/kg													
		50		500		20		3500				50			
		The first (March) and second (May) sampling campaign of 2015													
		March	May	March	May	March	May	March	May	March	May	March	May	March	May
Adamitulu Judo Kombolcha (AJK)	Edo Gojola	11.0	0.2	19.0	31.0	0.4	15.0	1.7	1.8	1.0	0.3	1.5	0.6	3.1	0.5
	Abune Germama	9.0	49.0	46.0	250	0.3	9.4	2.1	ND	3.7	0.7	0.6	4.9	0.8	15.0
	Bocessa	2.1	14.0	9.8	36.0	2.2	0.35*	4.4	0.3	13.0	0.04	0.5	4.5	0.4	13.0
	Doodicha	1.6	0.22*	14.0	< 2.7	0.3	4.1	3.4	5.9	19.0	< 0.4	1.5	0.2	1.9	0.2
	Ilika Chelema	27.0	7.1	120	33.0	4.5	5.0	6.1	14.0	11.0	1.6	0.7	0.3	1.2	0.2
Dugda Bora (DB)	Welda Mekedela	7.1	0.8	33.0	15.0	5.0	6.0	15.0	9.4	1.6*	3.7	0.2	0.5	0.3	1.0
	Welda Kelina	30.0	3.3	340	110	**130**	8.0	160	6.9	6.7*	4.2	13*	0.3	26*	0.3
	Shubi Gamo	0.09*	0.05*	5.9*	19.0	5.8	9.9	9.3	5.8	0.24*	6.8	0.3	0.3	0.2	0.2
	Bekeli Girisa	0.15*	0.24*	**810**	< 5.1	11.0	7.9	21.0	11.0	33.0	10.0	0.5	0.2	0.6	0.2
	Garba Korke Adi	4.4	0.7	**560**	14.0	5.9	2.8	2.8	10.0	57.0	0.25*	1.8	0.2	0.4	0.2
Arsi Negele (AN)	Shorba	0.05*	< 0.07	1.6*	5.0*	4.4	7.6	4.8	14.0	1.0	< 0.43	0.10	0.13	0.17	0.18
	Hargeda	0.09*	< 0.06	4.9*	4.6*	3.7	2.4	5.2	7.1	< 36	< 0.39	0.12	0.18	0.16	0.16
	Kerara	0.08*	< 0.11	< 1.9	7.3*	6.9	3.5	18.0	13.0	5.3*	12.0	0.15	0.16	0.17	0.20
	Gedemso	**64**	< 0.07	540	3.7*	3.0	3.9	3.4	11.0	0.29*	< 0.45	0.21	0.09	0.23	0.20
	Turge	0.17*	0.08*	6.6*	< 2.6	4.5	2.8	13.0	5.1	1.4*	< 0.44	0.14	0.14	0.24	0.18
Ziway Gugda (ZG)	Burka Lemefa	0.1*	0.07*	52.0	< 3.1	4.2	2.70	62.0	6.6	4.7	5.8	0.11	0.11	0.16	0.15
	Sengo	0.2*	0.19*	< 6.1	< 8.1	5.4	3.8*	31.0	9.0	4.2	11.0	0.08	0.15	0.16	0.32
	Galbe	< 0.06	**350**	14.0	ND	6.5	**470**	14.0	130.0	2.6	160	0.14	17*	0.22	**58**
	Shenen Meja	0.4	0.07*	5.8*	18.0	4.2	5.50	4.2	10.0	0.45*	11.0	0.11	0.19	0.21	0.26
	Burka Dalecha	0.03*	0.13*	8.3	22.0	8.4	6.10	3.6	8.1	15.0	6.7	0.32	0.23	0.27	0.20

*Value between LOD and LOQ. *< X* value less than LOD(X), *ND* not detected

Values were bolded just to show that they are significantly high results recorded among all the values obtained for the studied matrix

#### Profenofos

Profenofos was detected in all onion samples analyzed, and 5% of the samples were found to exceed the European MRL of 50 μg/kg (European Commission Regulation No, 2005). Among the samples above MRL at Gedemso and Galbe during the first and the second sampling campaigns, a concentration up to 350 μg/kg was found at the latter location and campaign (Table 4) due to the same reason that profenofos might be applied almost exclusively.

#### Metalaxyl

Except at Galbe in the second sampling campaign, metalaxyl was detected in all the samples analyzed (Table 4). 7.5% of the samples during the first sampling campaign exceeded the MRL of 500 μg/kg (European Commission Regulation No, 2005). This could be the farmers’ lack of awareness to spray this pesticide formulation in appropriate concentration (Mengistie et al. 2017). Metalaxyl was also found at a concentration of 250 μg/kg in the second sampling due to the low-temperature condition (Kassie et al. 2012) in this campaign that results in the less volatilization of this pesticide.

#### λ-Cyhalothrin

λ-Cyhalothrin was detected in all onion samples, and 5% of them exceeded the MRL of 20 μg/kg (European Commission Regulation No, 2005). The residues found at Galbe and Welda Kelina were 6–20-fold higher than the safe residue limits (Table 4).

#### DDE and DDT

Except at Abune Germama for 4,4′-DDT during the second sampling campaign, both DDTs were found in all onion samples (Table 4). However, none of the residues found were above the Codex MRL of 3500 μg/kg (Codex Alimentarius Commission 2009). 4,4′-DDT and 4,4′-DDE were detected at concentrations of 130 μg/kg and 160 μg/kg, respectively, at Galbe in the second sampling campaign. This might be due to the translocation of DDTs from contaminated soil and water to the onions at this location. For the control of malaria in the first sampling campaign, a considerable concentration difference of DDT was also found (Table 4). The higher 4,4′-DDT to 4,4′-DDE ratio in some of the locations might be due to the recent applications of DDT and its biotransformation to the environment (Kumar and Mukherjee 2012;Tavares et al. 1999).

#### Endosulfan

Both α- and β-endosulfan were detected in all onion samples and 2.5% of the samples have a concentration above MRL of 50 μg/kg (European Commission Regulation No, 2005). Among the 40 tested onion samples, 10% had an α-/β-endosulfan ratio of 2:1 during first sampling and 5% for the second campaign. 22.5% of the samples showed an α-/β-endosulfan ratio between 1:2 and 1:1 during the first sampling campaign and 15% during the second sampling campaign. Two percent and 7.5% of the samples had an 1:1 endosulfan ratio during the first and the second sampling, respectively (Table 4). In general, concentrations above MRLs may have a negative impact on the health conditions of the local farmers and the consumers within the country (since these vegetables are widely distributed and transported).

### Pesticide residues in water samples

**Fig. 2:**
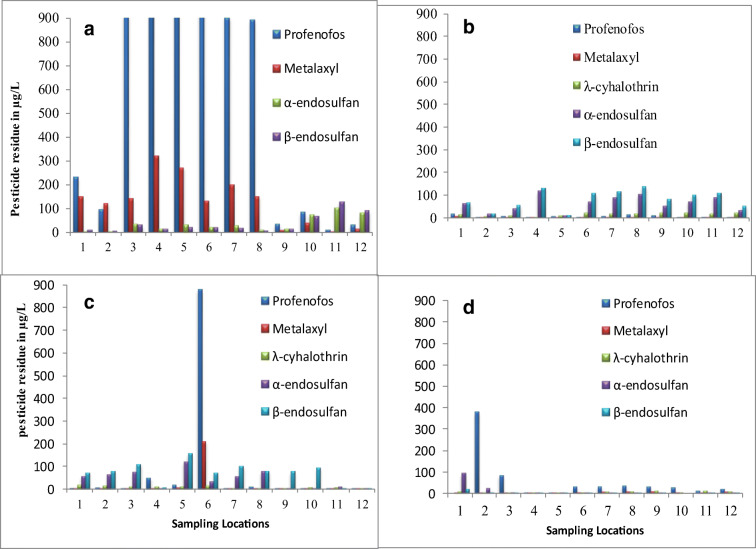
**a**–**d** CRV water sample pesticide concentrations in μg/L (sampling campaigns: **a** first sampling campaign (March of 2015), **b** second sampling campaign (May of 2015), **c** third sampling campaign (July of 2015), and **d** fourth sampling campaign (July of 2016)). Sample codes: 1 = HOLL, 2 = HILA, 3 = BOLZ, 4 = BILA, 5 = FE-1,6 = FE-2, 7 = BDW (Ziway), 8 = ANLZ-1, 9 = ANLZ-2, 10 = ANLZ-3, 11 = AALZ-1, and 12 = AALZ-2

Figure 2 summarizes the results of pesticide residues found in water samples in March and May of 2015, in July 2015, and in July of 2016. 4,4′-DDT and 4,4′-DDE residues were not found in water, which is obvious as these compounds have poor water solubility. Clearly, the levels of most pesticides were the highest in March (2015). This is true for the riverine and floriculture locations (nos. 1–6 in Fig. 2).

In particular, profenofos levels were high (up to 2300 μg/L in the Bulbula river), but also metalaxyl concentrations were high at most locations in the March (2015) campaign. Since there are more agricultural lands along this river, and this campaign took place during a short rainy season period (locally known as *Belg*) that receives a relatively low rain fall (175–358 mm) (Kassie et al. 2012), the pesticides could possibly run-off from these lands with less dilution effect in the river (Antic et al. 2015). Hence, this may have caused higher concentrations of profenofos and metalaxyl in this sampling campaign.

For the same reason, endosulfan and λ-cyhalothrin were higher in May and July of 2015, and the level of both endosulfan concentrations found was much higher than the Maximum Allowable Concentration (MAC = 0.01 μg/L) in rivers and lakes recommended by the European Union directive 2013/39 (European Union directive 2013). In the July (2016) campaign, all pesticides were found in lower concentrations (Fig. 2d).

The CRV received a high rainfall (420–680 mm) in this campaign, which is the main rainy season (locally known as *Kiremt*) (Kassie et al. 2012) and caused more dilution effect of the pesticide load in the rivers because of their high flow rate (Antic et al. 2015). This effect resulted in lower pesticide concentrations in the rivers during this period. The concentrations of endosulfans for all river locations and sampling campaigns in the present study were higher than in Densu River basin, Ghana (Kuranchie-Mensah et al. 2012), where α-endosulfan concentrations were found at a mean concentration of 0.025 μg/L, which is above the MAC of 0.01 μg/L.

Profenofos and metalaxyl were detected in higher concentrations in water samples collected from the agricultural lands (AALZ and ANLZ) in the March (2015) campaign (Fig. 2a). This is probably due to local traditions that require higher vegetable consumption during this time of the year. The farmers tend to apply higher volumes of pesticides then. The concentrations obtained in the water samples (above MAC) have a direct impact for the farmers since they use these water sources found in their lands, nearby rivers, and lakes for future vegetable productions, and also cause the contamination of the surrounding environments in the area. The metalaxyl concentrations in this study were much higher than those found in a similar study conducted in the CRV of Ethiopia (Jansen and Harmsen 2011) with concentrations ranging between 0.05 and 0.11 μg/L from different areas in both surface and drinking water samples. Although, recent data is not available for the amount of tomato and onion produced by the local farmers during this time of the year in our study areas. Putter et al. (2012) reported that 16,442 and 26,188 kg/ha of tomato and onion were produced by these farmers at AJK, respectively, and at the DB area, 33,699 kg/ha of tomato and 18,551 kg/ha of onion.

In effluents from the floriculture enterprises, profenofos was found at concentrations of 1700 μg/L and 880 μg/L in the first and third water sampling campaigns, respectively, and the metalaxyl concentration was also high up to 210 μg/L in third (Fig. 2a–c). These might come from the nearby Ziway Lake. However, in all other sampling locations, residues were less pronounced because these enterprises usually spray other groups of pesticides such as ethirimol and fenarimol (Jansen and Harmsen 2011) for flower and rose productions.

The pesticide concentrations in drinking water collected from tap at Batu Drinking Water (BDW) are also shown in Fig. 2. Profenofos and metalaxyl were detected in high concentration of 900 μg/L and 200 μg/L, respectively, during the first water sampling campaign (Fig. 2a). For the same reason, pressure on farming to meet the high demand for vegetables might have caused the pesticides entering into the drinking water supply at the Batu (Ziway) area which lacks a proper water treatment system. The lower pesticide concentrations in the third and fourth campaigns, both in July, are explained by a higher dilution in the rivers due to the heavy rain season in this period (Kassie et al. 2012). These rivers are used as a source of drinking water in the area.

## Conclusions

QuEChERS and SPE methods were successfully validated and used for the analysis of seven pesticides in tomatoes, onions, and water samples collected from the CRV of Ethiopia. Among these pesticides, profenofos and metalaxyl showed the highest concentrations for most of vegetables and water samples. The frequency of the detected pesticides in water samples was higher at Bulbula River than at Horankelo. At agricultural lands, in the first sampling campaign, pesticide levels were also high due to their intensive application during vegetable productions. The concentrations that exceed the MRLs in tomatoes and onions may cause major health problems to the farmers. Those and that exceed MAC in water from the two agricultural lands may cause damage to the local environment. Regular monitoring of pesticide levels in study samples and proper training and education on safe application of pesticides are crucial to reduce the potential health risks. Therefore, this study may serve as a basis for the concerned authorities in Ethiopia to take appropriate measures to make sure that the level of pesticide residues in different vegetables produced in the CRV stays below MRLs to protect the consumers and to reduce the exposure of local farmers and workers who are involved in pesticide applications.

## References

[CR1] Agarwal A, Pandey RS, Sharma B (2010). Water pollution with special reference to pesticide contamination in India. Journal of Water Resource and Protection.

[CR2] Agarwal A, Prajapati R, Singh OP, Raza SK, Thakur LK (2015). Pesticide residue in water-a challenging task in India. Environmental Monitoring and Assessment.

[CR3] Ahmed, M. A. H., Azhari, O. A., Abd Elaziz, S. A. I., Asma, A., & Mark, D. L. (2017). Determination of residues levels of seven pesticides in tomatoes samples taken from three markets in Khartoum State, Sudan *9*^*th*^*Int’l Conf. on Research in Chemical, Agricultural, Biological & Environmental Sciences* (RCABES-2017) Nov. 27–28, 2017 Parys, South Africa 10.17758/EARES.EAP517217

[CR4] Akca MO, Hisatomi S, Takemura M, Harada N, Nonaka M, Sakakibara F (2016). 4,4′-DDE and endosulfan levels in agricultural soils of the Cukurova Region, Mediterranean Turkey. Bulletin of Environmental Contamination and Toxicology.

[CR5] Akoto O, Gavor S, Appah MK, Apau J (2015). Estimation of human health risk associated with the consumption of pesticide-contaminated vegetables from Kumasi, Ghana. Environmental Monitoring and Assessment.

[CR6] Alemayehu T, Ayenew T, Kebede S (2006). Hydrogeochemical and lake level changes in the Ethiopian Rift Valley. Journal of Hydrology.

[CR7] Anastassiades MLS, Stajnbaher D, Schenck FJ (2003). Fast and easy multiresidue method employing acetonitrile extraction/partitioning and ‘dispersive solid-phase extraction’ for the determination of pesticide residues in produce. Journal of AOAC International.

[CR8] Angioni A, Porcu L, Dedola F (2011). Determination of famoxadone, fenamidone, fenhexamid and iprodione residues in greenhouse tomatoes. Pesticide Management Science.

[CR9] Antic N, Radisic M, Radovic T, Vasiljevic T, Grujic S, Petkovic A, Dimkic M, Lausevic M (2015). Pesticide residues in the Danube River Basin in Serbia - a survey during 2009–2011. Research Article. CLEAN- Soil, Air, Water.

[CR10] Antonious GF, Byers ME, Snyder JC (1998). Residues and fate of endosulfan on field-grown pepper and tomato. Pesticide Sciences.

[CR11] Arias LA, Bojacá CR, Ahumada DA, Schrevens E (2014). Monitoring of pesticide residues in tomato marketed in Bogota, Colombia. Food Control.

[CR12] Ayenew T (2004). Environmental implications of changes in the levels of lakes in the Ethiopian Rift since 1970. Regional environmental change.

[CR13] Berrada H, Fernandez M, Ruiz MJ, Molto JC, Manes J, Font G (2010). Surveillance of pesticide residues in fruits from Valencia during twenty months (2004/05). Food Control.

[CR14] Bhanti M, Taneja A (2005). Monitoring of organochlorine pesticide residues in summer and winter vegetables from Agra, India - a case study. Environmental Monitoring and Assessment.

[CR15] Camino-Sanchez FJ, Zafra-Gomez A, Ruiz-Garcia J, Bermu’dez-Peinado R, Ballesteros O, Navalon A (2011). UNE-EN ISO/IEC 17025:2005 accredited method for the determination of 121 pesticide residues in fruits and vegetables by gas chromatography–tandem mass spectrometry. Journal of Food Composition and Analysis.

[CR16] Chopra NM, Mahfouz AM (1977). Metabolism of endosulfan I, endosulfan II, and endosulfan sulfate on tobacco leaf. Journal of Agriculture and Food Chemistry.

[CR17] Codex Alimentarius Commission, (2009). Joint FAO/WHO Food Standards Programme:Distribution of the report of the forty-first session of the codex committee on pesticide residues (ALINORM 09/32/24) Beijing, China. 20–25 April 2009 . pp.1–116 http://www.crl-pesticides.eu/library/docs/fv/al32_24e.pdf. Accessed 01 October 2018.

[CR18] D’Mello, J. P. F. (2003). Food Safety: Contaminants and Toxins. *Scottish Agricultural College, Edinburgh, UK. April 2003, pp.1–455 Accessed*, (03 March 2017).

[CR19] Diop A, Diop YM, Thiaré DD, Cazier F, Sarr SO, Kasprowiak A (2016). Monitoring survey of the use patterns and pesticide residues on vegetables in the Niayes zone, Senegal. Chemosphere.

[CR20] EN 15662, (2008). Foods of plant origin-determination of pesticide residues using GC-MS and/or LC-MS/MS following acetonitrile extraction/partitioning and clean-up by dispersive SPE-QuEChERS method. pp. 1–84.http://www.chromnet.net/Taiwan/QuEChERS_Dispersive_SPE/QuEChERS_%E6%AD%90%E7%9B%9F%E6%96%B9%E6%B3%95_EN156622008_E.pdf. Accessed 2 November 2017

[CR21] Essumang DK, Dodoo DK, Adokoh CK, Fumador EA (2008). Analysis of some pesticide residues in tomatoes in Ghana. Human and Ecological Risk Assessment.

[CR22] Ethiopian Investment Agency (2012). Ethiopia Investment Guide.

[CR23] Ethiopian Press Agency and Floral Daily, (2016). The Ethiopian Horticulture Development Agency report, Addis Ababa Ethiopia. (On line document) http://www.intracen.org/itc/blogs/market-insider/Ethiopia-exports-225-million-USD-worth-of-cut-flowers/ Accessed 10 May 2020.

[CR24] European Commission Regulation No. 396/2005, (2005) European Parliament and of the Council of 23 February 2005 on maximum residue levels of pesticides in or on food and feed of plant and animal origin and amending Council Directive 91/ 414/EECText with EEA relevance. pp 1–2640. http://eur-lex.europa.eu/ LexUriServ/LexUriServ.do?uri¼CELEX:32005R0396:en:NOT. .

[CR25] European Union directive, (2013). Directive 2013/39/EU of the European parliament and of the council of 12 August 2013 amending Directives 2000/60/EC and 2008/105/EC as regards priority substances in the field of water policy. Accessed 05 April 2018.

[CR26] Fernandes VC, Domingues VF, Mateus N, Delerue-Matos C (2011). Organochlorine pesticide residues in strawberries from integrated pest management and organic farming. Journal of Agriculture and Food Chemistry.

[CR27] Fiedler H, Abad E, Van Bavel B, De Boer J, Bogdal C, Malisch R (2013). The need for capacity building and first results for the Stockholm Convention Global Monitoring Plan. Trends in Analytical Chemistry..

[CR28] Freixo JL, Dores EFGDC, Villa RD (2015). Sampling and analysis of pesticides in the gas phase of air: method validation using a volatilization chamber. Analytical Methods.

[CR29] Ghadiri H (2001). Degradation of endosulfan in a clay soil from cotton farms of western Queensland. Journal of Environmental Management.

[CR30] Goebel H, Gorbach S, Knauf W, Rimpau RH, Huttenbach H (1982). Properties, effects, residues and analytics of the insecticide endosulfan. Residue Review.

[CR31] Handford EC, Campbell K (2015). A review of the global pesticide legislation and the scale of challenge in reaching the global harmonization of food safety standards. Integrated Environmental Assessment and Management.

[CR32] Jansen, H. C., & Harmsen, J. (2011). Pesticide monitoring in the Central Rift Valley 2009–2010 ecosystems for water in Ethiopia Alterra-report 2083 Alterra, part of Wageningen UR, Wageningen.1–48. Accessed 19 April 2018.

[CR33] Jansen, H., Hengsdijk, H., Legesse, D., Ayenew, T., Hellegers, P., & Spliethoff, P. (2007). Land and water resources assessment in Ethiopia Central Rift Valley of Ethiopia. Alterra-rapport 1587. Alterra,Wageningen. pp. 1–83. Accessed 09 March 2017.

[CR34] Kassie, B. T., Rötter, R. P., Hengsdijk, H., Asseng, S., Van Ittersum, M. K., Kahiluoto, H., & Van Keulen, H. (2012). Climate variability and change in the Central Rift Valley of Ethiopia: challenges for rain-fed crop production. *Journal of Agricultural Sciences*, 1–17. 10.1017/S0021859612000986.

[CR35] Kathpal TS, Singh A, Dhankhar JS, Singh G (1997). Fate of endosulfan in cotton soil under subtropical conditions of Northern India. Pesticide Sciences.

[CR36] Kouzayha A, Rabaa AR, Iskandarani MA, Beh D, n Budzinski HL, Jaber F (2012). Multiresidue method for determination of 67 pesticides in water samples using solid-phase extraction with centrifugation and gas chromatography-mass spectrometry. American Journal of Analytical Chemistry.

[CR37] Kumar BM, Mukherjee DP (2012). Organochlorine residues in vegetables. International Journal of Vegetable Sciences.

[CR38] Kumari B, Madan VK, Kumar R, Kathpal TS (2002). Monitoring of seasonal vegetables for pesticide residues. Environmental Monitoring and Assessment.

[CR39] Kuranchie-Mensah H, Atiemo SM, Palm LMN-D, Blankson-Arthur S, Tutu AO, Fosu P (2012). Determination of organochlorine pesticide residue in sediment and water from the Densu river basin, Ghana. Chemosphere.

[CR40] Lehotay SJ, Son AK, Kwon H, Koesukwiwat U, Fu W, Mastovska K (2010). Comparison of QuEChERS sample preparation methods for the analysis of pesticide residues in fruits and vegetables. Journal of Chromatography A.

[CR41] Li-Ming, H., John, T., Albert, W., & Kean, G. (2008). Environmental chemistry, ecotoxicity, and fate of lambda-cyhalothrin. D.M. Whitacre (ed.), *Reviews of Environmental Contamination and Toxicology*, 1–21. 10.1007/978-0-387-77030-7_3 18418954

[CR42] Lu DS, Qiu XL, Feng CJY, Lin YJ, Xiong LB (2012). Simultaneous determination of 45 pesticides in fruit and vegetable using an improved QuEChERS method and on-line gel permeation chromatography-gas chromatography/mass spectrometer. Journal of Chromatography B.

[CR43] Mengistie BT, Mol AP, Oosterveer P (2017). Pesticide use practices among smallholder vegetable farmers in Ethiopian Central Rift Valley. A multidisciplinary approach to the theory and practice of sustainable development. Environment, Development and Sustainability.

[CR44] Mukherjee I, Gopal M (1994). Interconversion of stereoisomers of endosulfan on chickpea crop under field conditions. Pesticide Sciences.

[CR45] Mutengwe MT, Chidamba L, Korsten L (2016). Monitoring pesticide residues in fruits and vegetables at two of the biggest fresh produce markets in Africa. Journal of Food Protection.

[CR46] Osman KA, Al-Humaid AM, Al-Rehiayani SM, Al-Redhaiman KN (2010). Monitoring of pesticide residues in vegetables marketed in Al-Qassim region, Saudi Arabia. Ecotoxicology and Environmental Safety.

[CR47] Ozcan C (2016). Determination of organochlorine pesticides in some vegetable samples using GC-M. Polish Journal of Enviromental Studies.

[CR48] Parm PS, Raminderjit SB, Balwinder S, Rajinder LK (1991). Fate and interconversion of endosulfan I, II and sulfate on gram crop (*Cicer arietinum* Linn,) in subtropical environment. Bulletin of Environmental Contamination and Toxicology.

[CR49] Putter, H. D., Hengsdijk, H., Roba, S. T & Wayu, D. A. (2012). Scoping study of horticulture smallholder production in the Central Rift Valley of Ethiopia. Wageningen UR, Foundation Stichting Dienst Landbouwkundig Onderzoek (DLO) research institute Plant Research International and Ethiopian Horticultural Producer Exporters Association (EHPEA). pp. 1–64 https://edepot.wur.nl/249787 Accessed 12 December 2015.

[CR50] SANCO, (2011). Method validation and quality control procedures for pesticide residues analysis in food and feed. Directorate-General for Health and Consumers (DG SANCO), Brussels, Belgium. Document nr SANCO/12495/2011. pp. 1–41. http://ec.europa.eu/food/plant/protection/pesticides/docs/qualcontrol_en.pdf Accessed 12 March 2017.

[CR51] Scholten, W. (2007). Agricultural development and water use in the Central Rift Valley of Ethiopia: a rapid appraisal. *Project on Data collection for and analysis of smallholder farming in the Ethiopian Central Rift Valley. pp.*, 1–52 .

[CR52] Shunthirasingham C, Oyiliagu C, Cao X, Gouin T, Wania F, Lee SC (2010). Spatial and temporal pattern of pesticides in the global atmosphere. Journal of Enviromental Monitoring.

[CR53] Sneha D, Bhimte PU (2012). Meshram persistent organochlorine pesticide residues in ground and surface water of Nagpur and Bhandara district. Bionano Frontie.

[CR54] Srivastava AK, Trivedi P, Srivastava MK, Lohani M, Srivastava LP (2011). Monitoring of pesticide residues in market basket samples of vegetable from Lucknow City, India: QuEChERS method. Environmental Monitoring and Assessment.

[CR55] Stockholm Convention, (2001). Convention to Protect Human Health and Environment from the Harmful Impact of POPs. United Nations, Geneva, Switzerland. pp. 1–34. Accessed 22 June 2016.

[CR56] Sutherland TD, Horne I, Harcourt RL, Russell RJ, Oakeshott JG (2002). Isolation and characterization of a Mycobacterium strain that metabolizes the insecticide endosulfan. Journal of Applied Microbiology.

[CR57] Tadeo JL, Sanchez-Brunete C (2003). Analysis of pesticide residues in fruit juices by matrix-solid phase dispersion and gas chromatographic determination. Chromatographia.

[CR58] Tavares TM, Beretta M, Costa MC (1999). Ratio of DDT/DDE in the all saints bay, Brazil and its use in environmental management. Chemosphere.

[CR59] Teklu BM, Adriaanse PI, Ter Horst MMS, Deneer JW, Van den Brink PJ (2015). Surface water risk assessment of pesticides in Ethiopia. Sciences of the Total Environment.

[CR60] Walse SS, Scott GI, Ferry JL (2003). Stereoselective degradation of aqueous endosulfan in modular estuarine mesocosms: formation of endosulfan gamma-hydroxycarboxylate. Journal of Environmental Monitoring.

